# Association between *APOE, SCARB1, PPARα* polymorphisms and serum lipids in a population of Lithuanian adults

**DOI:** 10.1186/1476-511X-12-120

**Published:** 2013-08-06

**Authors:** Alina Smalinskiene, Janina Petkeviciene, Dalia Luksiene, Kristina Jureniene, Jurate Klumbiene, Vaiva Lesauskaite

**Affiliations:** 1 Laboratory of Molecular Cardiology, Institute of Cardiology, Medical Academy, Lithuanian University of Health Sciences, Kaunas, Lithuania; 2 Laboratory of Population Studies, Institute of Cardiology, Medical Academy, Lithuanian University of Health Sciences, Kaunas, Lithuania; 3 Faculty of Public Health, Medical Academy, Lithuanian University of Health Sciences, Kaunas, Lithuania

**Keywords:** Apolipoprotein E *(APOE)* genotype, Scavenger receptor class B type 1 (*SCARB1)* genotype, Peroxisome proliferator-activated receptor-alpha *(PPARα)* genotype, Dyslipidemia

## Abstract

**Background:**

Dyslipidemia is one of several known risk factors for coronary heart disease, a leading cause of death in Lithuania. Blood lipid levels are influenced by multiple genetic and environmental factors. Epidemiological studies demonstrated the impact of nutrition on lipid levels within the Lithuanian population although the role of genetic factors for dyslipidemias has not yet been studied. The objective of this study was to assess the distribution of the *APOE, SCARB1, PPARα* genotypes in the Lithuanian adult population and to determine the relationship of these genotypes with dyslipidemia.

**Methods:**

A cross-sectional health survey was carried out in a representative random sample of the Lithuanian population aged 25–64 (n=1030). A variety of single-nucleotide polymorphisms (SNPs) of the *APOE* (rs429358 and rs7412), *SCARB1* (rs5888) and *PPARα* (rs1800206) genes were assessed using real-time polymerase chain reaction. Serum lipids were determined using enzymatic methods.

**Results/Principal findings:**

Men and women with the *APOE2* genotype had the lowest level of total and low-density lipoprotein cholesterol (LDL-C). Men with the *APOE2* genotype had significantly higher levels of triglycerides (TG) than those with the *APOE3* genotype. In men, the carriers of the *APOE4* genotype had higher odds ratios (OR) of reduced (<1.0 mmol/L) high density lipoprotein cholesterol (HDL-C) levels versus *APOE3* carriers (OR=1.98; 95% CI=1.05-3.74). The odds of having elevated (>1.7 mmol/L) TG levels was significantly lower in *SCARB1* genotype CT carriers compared to men with the *SCARB1* genotype CC (OR=0.50; 95% CI=0.31-0.79). In men, carriers of the *PPARα* genotype CG had higher OR of elevated TG levels versus carriers of *PPARα* genotype CC (OR=2.67; 95% CI=1.15-6.16). The odds of having high LDL-C levels were lower in women with the *APOE2* genotype as compared to *APOE3* genotype carriers (OR=0.35; 95% CI=0.22-0.57).

**Conclusions/Significance:**

Our data suggest a gender difference in the associations between *APOE, SCARB1, PPARα* genotypes and lipid levels. In men, the *APOE4* genotype and *PPARα* genotype CG were correlated with an atherogenic lipid profile while the *SCARB1* genotype CT had an atheroprotective effect. In women, *APOE2* carriers had the lowest odds of high LDL-C.

## Background

A high prevalence of cardiovascular diseases is a major public health problem in Lithuania. In 2010, the age-standardized mortality from coronary heart disease (CHD) for Lithuanian men aged 25–64 years was 198.2 per 100 000 population and for women 44.0 per 100 000 population, while the average rate in the European Union was 102.0 per 100 000 population for men and 24.8 per 100 000 population for women of comparable age [1]. Dyslipidemia is one of several known risk factors for CHD. Blood lipid levels are influenced by multiple genetic and environmental factors [2].

Diet is the most important environmental determinant of lipid levels. Epidemiological studies have demonstrated some positive changes in the diet of the Lithuanian population over the last decades. The use of animal fats has declined, while the use of vegetable fats and the frequency of consumption of fresh vegetables have increased [3]. Consequently, the proportion of saturated fatty acids in the daily energy intake has decreased and the proportion of polyunsaturated fatty acids has increased [4]. Positive changes in diet have contributed to a decline in serum cholesterol levels; however, the mortality rate from CHD has remained high.

Several genes were found to be associated with lipid levels and the risk of cardiovascular diseases. Apolipoprotein E (APOE) is a glycoprotein that plays a fundamental role in lipid metabolism. APOE participates in the clearance of chylomicron remnants and very low-density lipoprotein (VLDL) by serving as a ligand for LDL receptors [5]. The APOE isoforms are coded by three alleles (ϵ2, ϵ3 and ϵ4) resulting in six different genotypes (ϵ2/2, ϵ2/3, ϵ2/4, ϵ3/3, ϵ3/4, and ϵ4/4) [6]. Many studies assessing the role of *APOE* polymorphism on plasma lipids have shown that the presence of the ϵ4 allele is associated with elevations in LDL-C, while the presence of ϵ2 is associated with decreased levels [7,8].

The human Class B Type I Scavenger Receptor (SCARB1) protein is a hepatic receptor with large affinity for HDL-C [9]. After HDL-C binds to SCARB1 in the liver, cholesterol is transferred to the cells to be released in the bile. Some prior studies of the effect of *SCARB1* genotypes on HDL-C have shown conflicting results. Studies among diabetics [10], CHD patients [11,12] and community-based populations [13] have reported that the *SCARB1* exon 8 rs5888 (C>T) polymorphism is associated with HDL-C. However, results of other studies have been inconsistent [14,15].

Peroxisome proliferator-activated receptor-a (PPAR*α*) is a member of the nuclear hormone receptor superfamily of ligand-inducible transcription factors and regulates the expression of genes involved in peroxisomal and mitochondrial ß-oxidation pathways such as fatty acid uptake and the catabolism of circulating triglycerides (TG) [16]. PPAR*α* is encoded by the *PPARα* gene located on chromosome 22q12-q13.1 [17]. It is expressed mainly in tissues demonstrating high capacity for fatty acid oxidation such as liver, kidney, heart, and skeletal muscle [18,19]. In studies of healthy subjects a relationship between the PPAR*α* L162V polymorphism and serum concentrations of TG [20,21] and LDL-C [22] has been suggested.

There is a lack of data regarding the role of genetic factors for lipid levels and cardiovascular risk in Lithuania. Our aim was therefore to assess the distribution of the *APOE, SCARB1, PPARα* genotypes in the Lithuanian adult population and to determine the relationship of these genotypes with dyslipidemia.

## Results

The characteristics of study subjects according to gender are given in Table 1. An association between lipid levels and gender was observed: men had lower levels of HDL-C and higher levels of TG compared to women. The prevalence of dyslipidemias was higher in men, except for a reduction in HDL, which was similar in both men and women. No significant differences in the frequencies of the *APOE, SCARB1* and *PPARa* genotypes or alleles between men and women were observed (Table 2). Frequencies of genotypes did not differ significantly from those predicted by a Hardy-Weinberg equilibrium.

**Table 1:** Characteristics of the study population

**Characteristics**	**Men**	**Women**	**p value**
Age, years	47.6 (0.51)	46.2 (0.43)	0.045
BMI, kg/m^2^ †	27.5 (0.26)	28.1 (0.22)	n.s.
TC, mmol/L ‡	5.32 (0.05)	5.30 (0.04)	n.s.
LDL-C, mmol/L ‡	3.40 (0.05)	3.21 (0.04)	0.002
HDL-C, mmol/L ‡	1.29 (0.02)	1.43 (0.02)	<0.001
LnTG, mmol/L ‡	0,39 (0,02)	0,31 (0,02)	0.001
High TC^#^, n (%)	253 (59.6)	278 (46.3)	<0.001
High LDL-C^#^, n (%)	265 (62.4)	279 (46.5)	<0.001
Reduced HDL^#^, n (%)	105 (24.7)	138 (22.9)	0.430
Elevated TG^#^, n (%)	140 (33.0)	136 (22.7)	<0.001
Obesity^#^, n (%)	105 (24.8)	151 (25.2)	n.s.
Diabetes^a,#^, n (%)	7 (1.7)	9 (1.5)	n.s.
Arterial hypertension^#^, n (%)	252 (59.4)	206 (34.3)	<0.001
Coronary heart disease^#^, n (%)	36 (8.6)	35 (5.9)	0.027

† Data adjusted by age.

‡ Data adjusted by age and BMI.

# Data weighted by age.

Data are presented as mean (standard error) unless otherwise indicated.

^a^Physician-diagnosed diabetes.

High TC level ≥5 mmol/L; high LDL-C level ≥3 mmol/L; reduced HDL-C level - for men <1.0 mmol/L and for women <1.2 mmol/L; elevated TG level >1.7 mmol/L.

*Abrevations: BMI* body mass index, *HDL-C* high density lipoprotein cholesterol, *LDL-C* low density lipoprotein cholesterol, *LnTG* logarithmically transformed triglycerides, *TC* total cholesterol.

**Table 2:** **Distribution of the *APOE, SCARB1, PPARα *genotypes and frequency of the alleles (%) in the study population**

**Characteristics**	**Men**	**Women**	**p value**
	**n (%)**	**n (%)**	
*APOE*,			n.s.
*ϵ 2/2* genotype	2 (0.5)	2 (0.3)	
*ϵ 3/2* genotype	76 (17.9)	94 (15.7)	
*ϵ 3/3* genotype	270 (63.6)	359 (59.7)	
*ϵ 4/2* genotype	13 (3.0)	21 (3.5)	
*ϵ 4/3* genotype	61 (14.5)	116 (19.3)	
*ϵ 4/4* genotype	2 (0.5)	9 (1.5)	
ϵ3 allele	79.8	77.2	n.s.
ϵ2 allele	11.0	9.9	
ϵ4 allele	9.2	12.9	
*SCARB1*,			n.s.
CC genotype	149 (35.2)	215 (35.8)	
CT genotype	212 (50,0)	287 (47.8)	
TT genotype	63 (14.8)	99 (16.4)	
C allele	58.9	58.4	n.s.
T allele	41.1	41.6	
*PPARα*,			n.s.
CC genotype	399 (94.2)	568 (94.5)	
CG genotype	25 (5.8)	32 (5.3)	
GG genotype	0	1(0.2)	
C allele	97.1	97.3	n.s.
G allele	2.90	2.70	

*Abrevations: APOE* apolipoprotein E, *PPARα* Peroxisome proliferator-activated receptor-alpha, *SCARB1* Scavenger Receptor Class B Type 1.

The fasting serum lipid levels of the study population with respect to the *APOE, SCARB1, PPARα* genotypes are shown in Table 3. Men and women with the *APOE2* genotype had the lowest level of TC and LDL-C. Mean values of TC and LDL-C were the highest in men and women with the *APOE4* genotype; however, they did not differ significantly from the mean values of those lipids in the *APOE3* genotype carriers. Men with the *APOE2* genotype had significantly higher levels of TG than those with the *APOE3* genotype. There were no significant differences in the levels of TG in women and in the levels of HDL-C in either gender among different genotype groups.

**Table 3:** **The fasting serum lipid levels in mmol/L (mean and standard error) according to the *APOE, SCARB1, PPARα *genotypes**

**The genotypes**	** TC**	** LDL-C**	** HDL-C**	** LnTG**
	** Mean (SE)**	** Mean (SE)**	**Mean (SE)**	** Mean (SE)**
		** MEN**		
***APOE***				
*APOE3*	5.38 (0.06)	3.49 (0.06)	1.30 (0.03)	0.35 (0.02)
*APOE2*	5.03 (0.12)*	3.00 (0.11)*	1.24 (0.05)	0.47 (0.04)*
*APOE4*	5.56 (0.13)**	3.60 (0.12)**	1.34 (0.05)	0.44 (0.05)
***SCARB 1***				
CC	5.30 (0.09)	3.29 (0.08)	1.26 (0.04)	0.45 (0.03)
CT	5.29 (0.07)	3.40 (0.07)	1.32 (0.03)	0.33 (0.03) ^#^
TT	5.56 (0.13)	3.66 (0.12) ^#^	1.27 (0.05)	0.39 (0.05)
***PPARα***				
CC	5.35 (0.05)	3.42 (0.05)	1.30 (0.02)	0.37 (0.02)
CG	5.31 (0.21)	3.33 (0.19)	1.29 (0.09)	0.58 (0.08)^# #^
		** WOMEN**		
***APOE***				
*APOE3*	5.34 (0.05)	3.31 (0.05)	1.42 (0.02)	0.31 (0.02)
*APOE2*	5.04 (0.10)*	2.74 (0.09)*	1.48 (0.04)	0.30 (0.04)
*APOE4*	5.39 (0.09)**	3.35 (0.08)**	1.45 (0.03)	0.30 (0.03)
***SCARB 1***				
CC	5.31 (0.07)	3.27 (0.06)	1.42 (0.03)	0.30 (0.03)
CT	5.29 (0.06)	3.23 (0.05)	1.42 (0.02)	0.32 (0.02)
TT	5.25 (0.10)	3.09 (0.09)	1.49 (0.04)	0.29 (0.04)
***PPARα***				
CC	5.28 (0.04)	3.21 (0.04)	1.42 (0.03)	0.31 (0.02)
CG	5.54 (0.18)	3.43 (0.16)	1.41 (0.02)	0.36 (0.07)

Data adjusted by age and body mass index.

*APOE2* - carriers of the ϵ 2/2 and ϵ 2/3 genotype, *APOE3* - carriers of the ϵ 3/3 genotype, and *APOE4* - carriers of the ϵ 3/4 and ϵ 4/4 genotype.

* p <0.05 compared with *APOE3* genotype; ** p <0.05 compared with *APOE2* genotype; ^#^ p <0.05 compared with *SCARB1*genotype CC; ^##^ p <0.05 compared with *PPARα* genotype CC.

*Abrevations: APOE* apolipoprotein E, *HDL-C* high density lipoprotein cholesterol, *LDL-C* low density lipoprotein cholesterol, *LnTG* logarithmically transformed triglycerides, *PPARα* Peroxisome proliferator-activated receptor-alpha, *SE* Standard Error, *SCARB1* Scavenger Receptor Class B Type 1, *TC* total cholesterol.

In men, the *SCARB1* TT genotype carriers had a significantly higher levels of LDL-C than men with the *SCARB1* genotype CC (Table 3). Moreover, men with the *SCARB1* CT genotype had lower levels of TG in comparison to CC genotype carriers. No significant differences in the levels of HDL-C between different *SCARB1* genotype groups were found in men. Serum lipid levels were not associated with *SCARB1* genotypes in women.

Men with the *PPARα* genotype CG had higher levels of triglycerides than did CC genotype carriers (Table 3). In women, no significant differences in serum lipid levels were found between different *PPARα* genotypes.

Multivariate logistic regression analysis was performed to identify the associations of the *APOE, SCARB1, PPARα* genotypes with the prevalence of dyslipidemias in the study population (Table 4). After adjustment for age, body mass index, physical activity and alcohol consumption, the odds of having high TC and high LDL-C levels were lower in women with the *APOE2* genotype compared to *APOE3* genotype carriers (respectively OR=0.52; p=0.006 and OR=0.35; p<0.001). In men, the odds of having reduced HDL-C levels was statistically higher in *APOE4* genotype carriers than in those with the *APOE3* genotype (OR=1.98; p=0.035). The associations of the *SCARB1* and *PPARα* genotypes with dyslipidemia were found only in men (Table 4). The odds of having elevated TG levels was statistically lower in the SCARB1 genotype CT carriers as compared to men with the SCARB1 genotype CC (OR=0.50; p=0.003). In men, carriers of the *PPARα* genotype CG had higher OR of elevated TG levels versus carriers of the *PPARα* genotype CC (OR=2.67; p=0.022).

**Table 4:** **Odds ratios for the likelihood of dyslipidemias according to the *APOE*, *SCARB1, PPARα *genotypes in the study population**

**The genotypes**	**High TC**	**High LDL-C**	**Reduced HDL-C**	**Elevated TG**
	**OR (95% CI)**	**OR (95% CI)**	**OR (95% CI)**	**OR (95% CI)**
		** MEN**		
***APOE***				
*APOE3*	1	1	1	1
*APOE2*	0.60 (0.33-1.02)	0.60 (0.35-1.02)	1.56 (0.85-2.85)	1.36 (0.79-2.34)
*APOE4*	1.04 (0.58-1.88)	1.37 (0.74-2.54)	**1.98 (1.05-3.74)**	1.50 (0.84-2.67)
***SCARB 1***				
CC	1	1	1	1
CT	1.20 (0.77-1.84)	1.09 (0.69-1.72)	0.76 (0.45-1.26)	**0.50 (0.31-0.79)**
TT	1.67 (0.89-3.23)	1.91 (0.98-3.73)	0.75 (0.36-1.58)	0.66 (0.35-1.26)
***PPARα***				
CC	1	1	1	1
CG	1.12 (0.47-2.66)	0.79 (0.34-1.84)	0.52 (0.15-1.84)	**2.67 (1.15-6.16)**
		** WOMEN**		
***APOE***				
*APOE3*	1	1	1	1
*APOE2*	**0.52 (0.32-0.83)**	**0.35 (0.22-0.57)**	0.62 (0.34-1.11)	0.97 (0.57-1.66)
*APOE4*	0.84 (0.54-1.30)	0.96 (0.62-1.49)	0.78 (0.48-1.29)	1.10 (0.69-1.76)
***SCARB 1***				
CC	1	1	1	1
CT	1.18 (0.80-1.74)	1.14 (0.78-1.68)	1.43 (0.92-2.23)	1.16 (0.76-1.77)
TT	1.39 (0.83-2.33)	0.83 (0.50-1.37)	0.82 (0.43-1.53)	1.03 (0.58-1.80)
***PPARα***				
CC	1	1	1	1
CG	1.71 (0.77-3.80)	1.95 (0.86-4.43)	1.23 (0.54-2.84)	0.82 (0.34-1.96)

Data adjusted by age, body mass index, physical activity, and alcohol consumption.

*APOE2* - carriers of the ϵ 2/2 and ϵ 2/3 genotype, *APOE3* - carriers of the ϵ 3/3 genotype, and *APOE4* - carriers of the ϵ 3/4 and ϵ 4/4 genotype.

*Abrevations: APOE* apolipoprotein E, *Elevated TG* triglycerides level >1.7 mmol/L, *High TC* total cholesterol ≥5.0 mmol/L, *High LDL-C* low density lipoprotein cholesterol ≥3.0 mmol/L, *PPARα* Peroxisome proliferator-activated receptor-alpha, *Reduced HDL- C* high density lipoprotein cholesterol level for men<1.0 mmol/L and for women <1.20 mmol/L, *SCARB 1* Scavenger Receptor Class B Type 1.

## Discussion

The overall pattern of the allele frequency distribution analyzed in the Lithuanian population was similar to other European populations. Nevertheless, some differences in *APOE* allele frequencies were observed between Northern European countries and Lithuania. Namely, a lower prevalence of the *APOE ϵ2* allele and a higher prevalence of the *APOE ϵ4* allele was found in Finland (4% and 20% respectively) [23], Denmark (8% and 17% respectively) [24] and Sweden (7% and 17% respectively) [25], as compared to the Lithuanian population. Findings from 45 populations around the world have shown that the frequency of the *APOE ϵ4* allele appears to be higher in northern regions of Europe (the Nordic countries, Scotland, Germany, and the Netherlands) than in southern regions (Switzerland, Tyrol, France, Italy, and Spain) [26]. The distribution of the *APOE* allele in Lithuania was more comparable to Southern European populations. The studies demonstrated a lower risk of CHD in *ϵ2* carriers and a higher risk in *ϵ4* carriers as compared to *ϵ3* homozygotes [8,27,28]. Bearing in mind the facts above, only a modest risk of CHD should be expected in Lithuania, although the mortality from CHD in the population of Lithuania is one of the highest in Europe [1].

Many studies suggested that variation of the *APOE* gene was associated with variations of lipid levels [7,8,24,25,27]. In line with the findings of other researchers, we found that *APOE2* genotype carriers had the lowest levels of TC and LDL-C. The mechanism of this effect probably involves decreased conversion of VLDL into LDL observed in *ϵ2* carriers [5,6]. More efficient catabolism of chylomicrons and VLDL-remnants, increased intestinal cholesterol absorption, and reduced LDL-receptor activity was shown in *ϵ4* carriers resulting in higher levels of TC and LDL-C when compared to *ϵ2* and *ϵ3* allele carriers. Thus, a high frequency of the *ϵ2* allele would appear to predict a favorable lipid profile in the Lithuanian population. Moreover, positive changes in nutritional habits of Lithuanians occurred over the last decades of the post-communist transition period and has contributed to the decrease in mean TC and LDL-C levels [3,4]. However, the prevalence of other cardiovascular disease risk factors, such as hypertension, smoking and obesity, remains very high in Lithuania [29]. It is obvious, that the risk of cardiovascular disease is determined by multiple genetic and environmental factors, and the impact of one gene is very limited. On the other hand, men with the *APOE2* genotype had higher levels of TG than those with the *APOE3* genotype. It was revealed that APOE2 binds poorly to lipoprotein receptors leading to accumulation of chylomicron and VLDL-remnants [6]. The higher TG levels in *APOE2* genotype carriers may attenuate the cardioprotective effects of lower LDL-C [24]. The *APOE* genotype also affected HDL-C level. Our data showed the higher odds of reduced HDL-C levels in men with the *APOE4* genotype as compared to those with the *APOE3* genotype. Other studies also found lower HDL-C in *ϵ4* allele carriers [7,24,25].

Much less is known regarding the role of *SCARB1* in humans, although several studies have reported that the SNP of *SCARB1* (rs5888) is associated with the lipid profile and the development of CHD [9,12,13]. The frequencies of the *SCARB1* alleles in our population were similar to frequencies that have been reported for French and North American populations [30]. Our data indicated that in men the carriers of the *SCARB1* genotype CT had two-fold lower odds of high TG levels than the carriers of the genotype CC. The tendency of lower odds of high TG levels was shown also for the *SCARB1* genotype TT carriers. Insufficient numbers of individuals with the *SCARB1* genotype TT in the study population may probably explain why the odds did not reach statistical significance. Men with the *SCARB1* genotype TT had a higher level of LDL-C as compared to *SCARB1* genotype CC carriers. In contrast to our findings, Morabia A et al. found higher LDL-C in women with the T allele [9]. We did not observe any association between the *SCARB1* genotype and dyslipidemia in women. Several studies have shown that a rare T variant was associated with an atheroprotective lipid profile in men but not in women [9,13]. A previous study carried out in Lithuania revealed that men aged 65–74 who were carriers of the TT genotype had a higher level of HDL-C and a lower risk of myocardial infarction as compared to the CC genotype [31]. Gender and age-specific effects on blood lipid profiles were also demonstrated by other investigators [12,13,15]. Those studies highlighted a protective T allele effect on HDL-C. SCARB1 was identified as a physiologically important HDL receptor; however, it is also a multiligand receptor participating in the metabolism of other plasma lipoproteins [14]. The mechanism of the *SCARB1* SNP rs5888 effect on the lipid profile remains not finally determined because polymorphisms in this SNP does not lead to a change in the amino acid sequence of the SCARB1 protein [9,14].

The prevalence of the *PPARα* G allele in our population was lower as compared to the prevalence that has been reported in other studies [20,22,32]. The results from a Quebec study indicated that carriers of the G allele may be at increased risk of abdominal obesity, hypertriglyceridemia, and low HDL-C levels [20]. The Framingham Offspring Study found that the G allele was associated with increased plasma concentrations of TC, LDL-C, and apoB [22]. In our study, the *PPARα* CG genotypes were associated with elevated TG levels only in men. It is known that PPARα stimulates proteins of the transport and binding of fatty acids. Moreover, it regulates genes involved in fatty acid oxidation [16,19]. Taking this in mind it is reasonable to believe that the functional L162V polymorphism located in the DNA-binding domain of the *PPARα* gene may have an effect on lipid metabolism, particularly on TG levels [33].

### Study strength and limitations

A major strength of our study is an investigation of a nationally representative random sample of the Lithuanian population, allowing assessment of the frequencies of alleles related to lipid levels. However, this study had some limitations. The cross-sectional study design did not allow us to consider the effects of all possible genetic and environmental factors and their interactions on lipid levels. Another issue involves the unhealthy diet of the majority of the Lithuanian population. A high intake of fats, especially saturated fats, might influence lipid levels and have an important interaction with the genotypes under study. Furthermore, we did not have data about hormone replacement therapy in postmenopausal women which might also affect the association between genotypes and lipid levels.

## Conclusions

Our data suggested a gender difference of the associations between *APOE, SCARB1, PPARα* genotypes and lipid levels. In men, the *APOE4* genotype and the *PPARα* genotype CG were correlated with an atherogenic lipid profile while the *SCARB1* genotype CT had an atheroprotective effect on lipid levels. In women, *APOE2* carriers had the lowest odds of high LDL-C. Further studies, particularly evaluating genetic-environmental interactions on lipid levels, are necessary to confirm our established associations.

## Materials and methods

### Study design and sample

The cross-sectional health survey was carried out in five randomly selected municipalities of Lithuania. The random sample was obtained from lists of inhabitants aged 25–64. Health examinations were conducted for 1739 participants (58% of the eligible sample). From these, 1030 individuals (425 men and 605 women) had the *APOE, SCARB1* and *PPARα* genotypes determined. We excluded 5 subjects taking lipid lowering medications from our analysis (1 man and 4 women). Also we excluded 34 subjects (13 men and 21 women) with the rare *APOE* ϵ2/4 genotype from the analysis of associations between the *APOE* genotypes and lipid levels.

### *APOE, SCARB1* and *PPARα* genotyping

For DNA extraction, blood samples were collected from each individual in ethylenediaminetetraacetic (EDTA) tubes during their health examination. DNA was extracted from peripheral blood leukocytes using a reagent kit (NucleoSpin Blood L Kit; Macherey & Nagel, Düren, Germany).

Two single-nucleotide polymorphisms (SNPs) of *APOE* gene (rs429358 and rs7412) were assessed using commercially available genotyping kit C___3084793_20 and C____904973_10. SNP of SCARB1 gene (rs5888) was assessed using commercially available genotyping kit C_7497008_1_ and SNP of *PPARα* gene (rs1800206) was assessed using commercially available genotyping kit C___8817670_20 (Applied Biosystems, Foster City, CA, USA). We used the Applied Biosystems 7900HT Real-Time Polymerase Chain Reaction System for detecting the SNPs. The cycling program started with heating at 95°C for 10 min, followed by 40 cycles (at 95°C for 15 s and at 60°C for 1 min). Allelic discrimination was carried out using the software of Applied Biosystems.

Three *APOE* genotype groups were analyzed in this study: *APOE2* (carriers of the *ϵ 2/2* and *ϵ 2/3* genotype), *APOE3* (carriers of the *ϵ 3/3* genotype), and *APOE4* (carriers of the *ϵ 3/4* and *ϵ 4/4* genotypes).

### Laboratory analyses and anthropometric measurements

Blood samples for lipids measurements were taken in the morning after fasting at least 12 hours. TC, LDL-C, HDL-C, and TG levels were determined by an automatic analyzer using conventional enzymatic methods. All laboratory analyses were made in the same certified laboratory. Quality control measures were followed for estimation of lipid levels. Dyslipidemias were defined using the following criteria: high TC level - serum TC≥5 mmol/L; high LDL-C level - serum LDL-C ≥3 mmol/L; reduced HDL-C level - serum HDL-C for men <1.0 mmol/L and for women <1.2 mmol/L; elevated TG level- TG >1.7 mmol/L [34].

The height of participants wearing no shoes was measured to the nearest centimeter with a stadiometer. The body weight of participants wearing light indoor clothing and no shoes was measured to the nearest 0.1 kg with standardized medical scales. Body mass index (BMI) was calculated as weight divided by height squared (kg/m^2^). Obesity was defined as BMI≥30 kg/m^2^.

### Assessment of physical activity and alcohol consumption

Information on physical activity and alcohol consumption was gathered by a standard questionnaire. The number of hours spent per week for physical activity at work, travelling to and from work and at leisure time was calculated. The questionnaire on alcohol consumption contained questions about a type and frequency of alcohol consumption and amount of alcohol consumed at one occasion. The amount of alcohol consumed at one occasion was recalculated into standard alcohol units (SAUs) using the following formula: SAU = amount (in liters) × strength of alcoholic drink (beer – 5%, wine – 12%, strong alcohol – 40%). One SAU equals to 10 g of ethanol. Then the amount of SAUs consumed during a month was calculated.

### Statistical analysis

The data were analyzed with statistical software package SPSS version 19.0 for Windows. All data were analyzed in each gender separately. Continuous variables were presented as mean values and standard error (SE). The normality of distribution of continuous variables was tested by Kolmogorov - Smirnov test. Only distribution of triglyceride levels was skewed, and this variable was logarithmically transformed to improve normality for statistical testing. Analysis of variance with Bonferroni multiple comparison tests were used to compare the means of continuous variables across groups.

The mean values of TC, LDL-C, HDL-C and TG levels by the *APOE, SCARB1, PPARα* genotypes were calculated using general linear model and controlling for age, BMI, physical activity and alcohol consumption.

The effects of the *APOE, SCARB1, PPARα* genotypes on the prevalence of dyslipidemias were evaluated using logistic regression analysis. All models were applied separately for men and women. Each genotype was included into a separate model. The odds of dyslipidemias were calculated with adjustment for age, body mass index, physical activity and alcohol consumption.

### Ethics statement

The study protocol was approved by the Lithuanian Bioethics Committee. Written informed consent for participation in the study was obtained from all participants.

## Abbreviations

TC: Total cholesterol; LDL-C: Low density lipoprotein cholesterol; TG: Triglycerides; HDL-C: High density lipoprotein cholesterol; CHD: Coronary heart disease; APOE: Apolipoprotein E; VLDL: Very low-density lipoprotein; SCARB1: Scavenger receptor class B type 1; PPARα: Peroxisome proliferator-activated receptor-alpha; SNPs: Single nucleotide polymorphisms; BMI: Body mass index; SAUs: Standard alcohol units; SE: Standard error; OR: Odds ratio; CI: Confidence intervals.

## Competing interests

None of the authors has any proprietary interests or conflicts of interest related to this submission. This submission has not been published anywhere previously, and it is not simultaneously being considered for any other publication.

## Authors’ contributions

AS participated in the design of the study, has made analysis and interpretation of data; drafted the manuscript. JP participated in the design of the study, has made analysis and interpretation of data; revised manuscript critically for important intellectual content, has given final approval of the version to be published. DL has made analysis and interpretation of data; helped to draft the manuscript. KJ has made analysis and interpretation of data. JK has made substantial contributions to conception and design, coordinated study, participated in acquisition of data, revised manuscript critically for important intellectual content. VL has made substantial contributions to conception and design, revised manuscript critically for important intellectual content, has given final approval of the version to be published. All authors read and approved the final manuscript.

## References

[B1] European Health for All Database (HFA-DB) Available: http://www.euro.who.int/hfadb Accessed 12 May 2013

[B2] Rotondo D Davidson J Genetics and molecular biology: identification of dyslipidemia genes Curr Opin Lipidol 2010 21 548 549 10.1097/MOL.0b013e3283404fde 21206343

[B3] Kriaucionienė V Klumbiene J Petkeviciene J Sakyte E Time trends in social differences in nutrition habits of a Lithuanian population:1994–2010 BMC Public Health 2012 12 218 10.1186/1471-2458-12-218 22436087 PMC3323430

[B4] Ramazauskiene V Petkeviciene J Klumbiene J Kriaucioniene V Sakyte E Diet and serum lipids: changes over socio-economic transition period in Lithuanian rural population BMC Public Health 2011 11 447 10.1186/1471-2458-11-447 21651754 PMC3128027

[B5] Mahley RW Innerarity TL Rall SC Jr Weisgraber KH Plasma lipoproteins: apolipoprotein structure and function J Lipid Res 1984 25 1277 1294 6099394

[B6] Weisgraber K Apolipoprotein E: structure-function relationships Adv Protein Chem 1994 45 249 302 8154371 10.1016/s0065-3233(08)60642-7

[B7] Kofler BM Miles EA Curtis P Armah CK Tricon S Grew J Napper FL Farrell L Lietz G Packard CJ Caslake MJ Mathers JC Williams CM Calder PC Minihane AM Apolipoprotein E genotype and the cardiovascular disease risk phenotype: impact of sex and adiposity (the FINGEN study) Atherosclerosis 2012 221 467 470 10.1016/j.atherosclerosis.2012.01.042 22365656

[B8] Ward H Mitrou PN Bowman R Luben R Wareham NJ Khaw KT Bingham S APOE genotype, lipids, and coronary heart disease risk Arch Intern Med 2009 69 1424 1429 19667307 10.1001/archinternmed.2009.234

[B9] Morabia A Ross BM Costanza MC Cayanis E Flaherty MS Alvin GB Das K James R Yang AS Evagrafov O Gilliam TC Population-based study of SR-BI genetic variation and lipid profile Atherosclerosis 2004 175 159 168 10.1016/j.atherosclerosis.2004.03.014 15186961

[B10] McCarthy JJ Lewitzky S Reeves C Permutt A Glaser B Groop LC Lehner T Meyer JM Polymorphisms of the HDL receptor gene associated with HDL cholesterol levels in diabetic kindred from three populations Hum Hered 2003 55 163 170 10.1159/000073986 14566094

[B11] McCarthy JJ Lehner T Reeves C Moliterno DJ Newby LK Rogers WJ Topol EJ Association of genetic variants in the HDL receptor, SR-BI, with abnormal lipids in women with coronary artery disease J Med Genet 2003 40 453 458 10.1136/jmg.40.6.453 12807968 PMC1735488

[B12] Rodríguez-Esparragón F Rodríguez-Pérez JC Hernández-Trujillo Y Macias-Reyes A Medina A Caballero A Ferrario CM Allelic variants of the human scavenger receptor class B type 1 and paraoxonase 1 on coronary heart disease. genotype-phenotype correlations Arterioscler Thromb Vasc Biol 2005 25 854 860 10.1161/01.ATV.0000157581.88838.03 15681296

[B13] Osgood D Corella D Demissie S Cupples LA Wilson PWF Meigs JB Schaefer EJ Coltell O Ordovas JM Genetic variation at the Scavenger receptor class B type I gene locus determines plasma lipoprotein concentrations and particle size and interacts with type 2 diabetes: the Framingham study J Clin Endocrinol Metab 2003 88 2869 2879 10.1210/jc.2002-021664 12788901

[B14] Acton S Osgood D Donoghue M Corella D Pocovi M Cenarro A Mozas P Keilty J Squazzo S Woolf EA Ordovas JM Association of polymorphisms at the SR-BI gene locus with plasma lipid levels and body mass index in a white population Arterioscler Thromb Vasc Biol 1999 19 1734 1743 10.1161/01.ATV.19.7.1734 10397692

[B15] Roberts CG Shen H Mitchell BD Damcott CM Shuldiner AR Rodriguez A Variants in scavenger receptor class B type I gene are associated with HDL cholesterol levels in younger women Hum Hered 2007 64 107 113 10.1159/000101962 17476110 PMC2861530

[B16] Schoonjans K Staels B Auwerx J Role of the peroxisome proliferator-activated receptor (PPAR) in mediating the effects of fibrates and fatty acids on gene expression J Lipid Res 1996 37 907 925 8725145

[B17] Sher T Yi HF McBride OW Gonzalez FJ cDNA cloning, chromosomal mapping, and functional characterization of the human peroxisome proliferator activated receptor Biochemistry 1993 32 5598 5604 10.1021/bi00072a015 7684926

[B18] Braissant O Foufelle F Scotto C Dauca M Wahli W Differential expression of peroxisome proliferator-activated receptors (PPARs): tissue distribution of PPAR-a, -b, and -c in the adult rat Endocrinology 1996 137 354 366 10.1210/en.137.1.354 8536636

[B19] Auboeuf D Rieusset J Fajas L Vallier P Frering V Riou JP Staels B Auwerx J Laville M Vidal H Tissue distribution and quantification of the expression of mRNAs of peroxisome proliferators activated receptors and liver Xreceptor-alpha in humans: no alteration in adipose tissue of obese and NIDDM patients Diabetes 1997 46 1319 1327 10.2337/diabetes.46.8.1319 9231657

[B20] Robitaille J Brouillette C Houde A Lemieux S Pe´russe L Tchernof A Gaudet D Vohl MC Association between the PPAR*α*- L126V polymorphism and components of the metabolic syndrome J Hum Genet 2004 49 482 489 15309680 10.1007/s10038-004-0177-9

[B21] Nielsen E-MD Hansen L Echwald SM Drivsholm T Borch- Johnsen K Ekstrøm CT Hansen T Pedersen O Evidence for an association between the Leu162Val polymorphism of the PPAR*α* gene and decreased fasting serum triglyceride levels in glucose tolerant subjects Pharmacogenetics 2003 13 417 423 10.1097/00008571-200307000-00007 12835617

[B22] Tai ES Demissie S Cupples LA Corella D Wilson PW Schaefer EJ Ordovas JM Association between the PPARA L162V polymorphism and plasma lipid levels: the Framingham offspring study Arterioscler Thromb Vasc Biol 2002 22 805 810 10.1161/01.ATV.0000012302.11991.42 12006394

[B23] Grönroos P Raitakari OT Kähönen M Hutri-Kähönen N Marniemi J Viikari J Lehtimäki T Relation of apolipoprotein E polymorphism to markers of early atherosclerotic changes in young adults–the cardiovascular risk in young finns study Clin Chem Lab Med 2008 46 179 186 18324906 10.1515/CCLM.2008.033

[B24] Frikke-Schmidt R Tybjaerg-Hansen A Steffensen R Jensen G Nordestgaard BG Apolipoprotein E genotype: epsilon32 women are protected while epsilon43 and epsilon44 men are susceptible to ischemic heart disease: the Copenhagen city heart study J Am Coll Cardiol 2000 35 1192 1199 10.1016/S0735-1097(00)00520-9 10758960

[B25] Gustavsson J Mehlig K Leander K Strandhagen E Björck L Thelle DS Lissner L Blennow K Zetterberg H Nyberg F Interaction of apolipoprotein E genotype with smoking and physical inactivity on coronary heart disease risk in men and women Atherosclerosis 2012 220 486 492 10.1016/j.atherosclerosis.2011.10.011 22071360

[B26] Gerdes LU Klausen C Sihm I Faergeman O Vogler GP Apolipoprotein E polymorphism in a Danish population compared to findings in 45 other study populations around the world Genet Epidemiol 1992 9 155 167 10.1002/gepi.1370090302 1381696

[B27] Bennet AM Di Angelantonio E Ye Z Wensley F Dahlin A Ahlbom A Keavney B Collins R Wiman B de Faire U Danesh J Association of apolipoprotein E genotypes with lipid levels and coronary risk JAMA 2007 298 1300 1311 10.1001/jama.298.11.1300 17878422

[B28] Song Y Stampfer MJ Liu S Meta-analysis: apolipoprotein E genotypes and risk for coronary heart disease Ann Intern Med 2004 141 137 147 10.7326/0003-4819-141-2-200407200-00013 15262670

[B29] Grabauskas V Klumbiene J Petkeviciene J Petrauskiene A Tamosiunas A Kriaucioniene V Ramazauskiene V Risk factors for non-communicable diseases in Lithuania rural population: CINDI survey 2007 Medicina 2008 44 633 639 18791341

[B30] Zerbib J Seddon JM Richard F Reynolds R Leveziel N Benlian P Borel P Feingold J Munnich A Soubrane G Kaplan J Rozet JM Souied EH rs5888 variant of SCARB1 gene is a possible susceptibility factor for age-related macular degeneration PLoS One 2009 5;4 7341 19806217 10.1371/journal.pone.0007341 PMC2752725

[B31] Stanislovaitiene D Lesauskaite V Zaliuniene D Smalinskiene A Gustiene O Zaliaduonyte-Peksiene D Tamosiunas A Luksiene D Petkeviciene J Zaliunas R SCARB1 single nucleotide polymorphism (rs5888) is associated with serum lipid profile and myocardial infarction in an age- and gender-dependent manner Lipids Health Dis 2013 Mar 5 12 24 10.1186/1476-511X-12-24 23510561 PMC3599926

[B32] Vohl MC Lepage P Gaudet D Brewer CG Bétard C Perron P Houde G Cellier C Faith JM Després JP Morgan K Hudson TJ Molecular scanning of the human PPARa gene: association of the L162V mutation with hyperapobetalipoproteinemia J Lipid Res 2000 41 945 952 10828087

[B33] Sparso T Hussain M Andersen G Hainerova I Borch-Johnsen K Jorgensen T Hansen T Pedersen O Relationships between the functional PPARa Leu162Val polymorphism and obesity, type 2 diabetes, dyslipidaemia, and related quantitative traits in studies of 5799 middle-aged white people Mol Genet Metab 2007 90 205 209 10.1016/j.ymgme.2006.10.007 17129741

[B34] Perk J De Backer G Gohlke H Graham I Reiner Z Verschuren M Albus C Benlian P Boysen G Cifkova R Deaton C Ebrahim S Fisher M Germano G Hobbs R Hoes A Karadeniz S Mezzani A Prescott E Ryden L Scherer M Syvänne M Scholte op Reimer WJ Vrints C Wood D Zamorano JL Zannad F European Guidelines on cardiovascular disease prevention in clinical practice (version 2012) Eur Heart J 2012 33 1635 1701 22555213 10.1093/eurheartj/ehs092

